# Quantifying the Quality of Medical Education Studies: The MMERSQI Approach

**DOI:** 10.1186/s12909-023-04303-3

**Published:** 2023-05-08

**Authors:** Aaron Lawson McLean, Falko Schwarz

**Affiliations:** Department of Neurosurgery, Jena University Hospital – Friedrich Schiller University Jena, Am Klinikum 1, 07747 Jena, Germany

Dear Editor,

We read with interest the recent publication "A Modified Medical Education Research Study Quality Instrument (MMERSQI) developed by Delphi Consensus" by Al Asmri et al. [1]. The authors present a modification of a commonly used study quality instrument, the Medical Education Research Study Quality Instrument (MERSQI), using a modified Delphi technique to reach consensus among experts in the field of medical education, with the aim of identifying any changes required in the scoring system and relative importance of each domain [2]. The authors also added new criteria to the instrument based on feedback received from the Delphi panel. The final criteria list and the new domain weighting score of the MMERSQI was satisfactory to all respondents and the authors suggest that the MMERSQI may help establish a reference standard of quality measures for many medical education studies.

The development of this tool is an important step in the evaluation of medical education research studies and the fact that that the authors obtained high levels of agreement among the Delphi panel members is a strength of the study, suggesting that the MMERSQI has good face validity. However, while the MMERSQI, like the original MERSQI, has certain strengths, we have concerns that need to be addressed before the tool can be widely adopted and used.

First, we appreciate the use of the Delphi method in reaching a consensus on the MMERSQI items. This method helps to ensure that the resulting instrument reflects the opinions of experts in the field and minimizes the risk of bias. However, the sample size of the Delphi participants could have been larger and more representative of the field, which would have increased the external validity of the results. Moreover, the specific criteria used to select the Delphi participants and the method of data analysis were not reported in the article, which limits the transparency and reproducibility of the study.

Second, the MMERSQI only covers a limited number of aspects of study quality. While the items selected are important, they do not encompass all the aspects of quality that need to be considered when evaluating medical education research studies. For example, ethical considerations like obtaining informed consent from participants, protecting participant confidentiality, and minimizing harm should also be included. Additionally, confounding variables, which can impact the validity of the results, should be reported.

Third, the scoring system used in the MMERSQI is not optimal. In the study, the weighting system was determined through a Delphi consensus, which while useful in gaining a general consensus, may not accurately reflect the true importance of each item in relation to the quality of the study. Further, the scoring system operates on a binary principle, in which a score is assigned based on the presence or absence of certain key features, without considering the importance of these features relative to one another. The weight given to each criterion is not well justified, nor is the weighting system anchored to a clear definition of study quality or to a clear understanding of the importance of each item in determining the quality of a study. This lack of weighting can lead to a flawed representation of study quality, as critical components may be given equal consideration as less critical ones. This issue is compounded by the fact that some components, such as participant characteristics or response rate, can have a significant impact on the overall validity of the study, whereas others, such as the type of institution, may have a relatively minor impact. Thus, the scoring system as it stands may not accurately reflect the true quality of the study, which can hinder the interpretation of the results and their application in practice.

It would have been helpful if the authors had provided some guidelines or a scoring algorithm to assist in the interpretation of the scores. The current approach does not allow for a nuanced assessment of the quality of the study and may result in a misleading overall score. A more nuanced scoring system, such as a modified Likert scale, could have been used to allow for a more nuanced assessment of the quality of each item. This would have allowed for a more in-depth examination of the strengths and limitations of each study, and would have enabled a more accurate and comprehensive assessment of study quality. Moreover, the scoring system could also be enhanced by considering the interplay between components, as certain combinations of features may have a compounded impact on the overall validity of the study. For example, a study with a low response rate may be compensated for by the use of high-fidelity simulation for measuring outcomes, whereas a study with a high response rate but limited internal validity may still produce questionable results.

Finally, the MMERSQI needs to be further tested and validated before it can be used in practice. The authors reported moderate to high levels of agreement among the Delphi panel members, but this does not necessarily mean that the MMERSQI is a valid and reliable instrument. Psychometric testing of the instrument, encompassing features such as reliability, validity and responsiveness, must now be pursued. Furthermore, the generalizability of the MMERSQI to different medical education research study designs and settings has not been established. A possible research agenda would include testing the MMERSQI in a range of contexts, assessing its reliability by measuring inter-rater agreement, and validating it against external standards of study quality, including alternative instruments like the Newcastle-Ottawa Scale-Education (NOS-E) or expert evaluations [3].

Of course, we recognise that obtaining expert evaluations or using a validated instrument for comparison can be time-consuming, costly, and may not always be feasible for every manuscript or study. Moreover, the use of an external gold standard assumes a level of consistency in the ratings provided, which may not always be the case. Nevertheless, efforts to develop and validate the MMERSQI should aim to incorporate as much external validation as possible, to improve its representativeness, precision in scoring, and the configuration of its multiple components, within the constraints of practicality and available resources.

In conclusion, while the MMERSQI is a useful step in the evaluation of medical education research studies, it still has several limitations and needs to be further developed and tested. It is clear to us that several of our comments, for example regarding the scoring procedure, selection of assessment criteria and dimensions, and interpretation of these attributes as indicators of study validity are fundamental issues that require attention in both the original MERSQI and the newly developed MMERSQI. We recommend that the authors conduct additional psychometric testing, validate the MMERSQI in different study designs and settings, and consider adding items to address the ethical and reporting aspects of study quality. We hope that the authors will take these aspects into consideration and that the MMERSQI will be further developed and refined to provide a comprehensive and accurate evaluation of medical education research studies.

Sincerely,

Aaron Lawson McLean & Falko Schwarz

## Data Availability

Not applicable.
